# An mHealth Physical Activity Intervention for Latina Adolescents: Iterative Design of the Chicas Fuertes Study

**DOI:** 10.2196/26195

**Published:** 2021-06-15

**Authors:** Britta Larsen, Emily D Greenstadt, Brittany L Olesen, Bess H Marcus, Job Godino, Michelle M Zive

**Affiliations:** 1 Herbert Wertheim School of Public Health and Human Longevity Science University of California San Diego San Diego, CA United States; 2 Department of Behavioral and Social Sciences Brown University Providence, RI United States; 3 Department of Pediatrics University of California San Diego San Diego, CA United States

**Keywords:** mobile health, human-centered design, qualitative research, adolescent health, health disparities, mobile phone

## Abstract

**Background:**

Only 3% of Latina teens meet the national physical activity (PA) guidelines, and these habits appear to persist into adulthood. Developing effective interventions to increase PA in Latina teens is necessary to prevent disease and reduce disparities. Mobile technologies may be especially appropriate for this population, but mobile health (mHealth) intervention content must be designed in collaboration with the target population.

**Objective:**

This study aims to develop an mHealth PA intervention for Latina adolescents using a multistage iterative process based on the principles of human-centered design and multiple iterations of the design phase of the IDEAS (Integrate, Design, Assess, Share) framework.

**Methods:**

On the basis of the feedback from a previous pilot study, the planned intervention included visual social media posts and text messaging, a commercial wearable tracker, and a primarily visual website. The development of the requested mHealth intervention components was accomplished through the following 2 phases: conducting focus groups with the target population and testing the usability of the final materials with a youth advisory board (YAB) comprising Latina adolescents. Participants for focus groups (N=50) were girls aged 13-18 years who could speak and read in English and who were recruited from local high schools and after-school programs serving a high proportion of Latinos. Facilitated discussions focused on experience with PA and social media apps and specific feedback on intervention material prototypes and possible names and logos. Viable products were designed based on their feedback and then tested for usability by the YAB. YAB members (n=4) were Latinas aged 13-18 years who were not regularly active and were recruited via word of mouth and selected through an application process.

**Results:**

The focus group discussions yielded the following findings: PA preferences included walking, running, and group fitness classes, whereas the least popular activities were running, swimming, and biking. Most participants (n=48, 96%) used some form of social media, with Instagram being the most favored. Participants preferred text messages to be sent no more than once per day, be personalized, and be positively worded. The focus group participants preferred an intervention directly targeting Latinas and social media posts that were brightly colored, included girls of all body types, and provided specific tips and information. Modified intervention materials were generally perceived favorably by the YAB members, who provided suggestions for further refinement, including the shortening of texts and the incorporation of some Spanish phrases.

**Conclusions:**

Latina teens were generally enthusiastic about an mHealth PA intervention, provided that the materials were targeted specifically to them and their preferences. Through multiple iterations of development and feedback from the target population, we gained insight into the needs of Latina teens and joined with industry partners to build a viable final product.

## Introduction

### Adolescent Girls’ Physical Activity

Adolescent girls report the lowest levels of regular physical activity (PA), particularly girls from racial and ethnic minority backgrounds [1]. Although only 8% of adolescents meet the national guidelines of 60 minutes of moderate-to-vigorous physical activity (MVPA) per day [2], the number is even worse when examined by gender and ethnicity, with only 2.9% of Mexican American adolescent girls meeting the guidelines (compared with 17.9% of Mexican American boys) [1]. These disparities in adolescents are paralleled by disparities in adulthood, with Latina women reporting less MVPA than non-Latino White and non-Latino Black women and higher rates of lifestyle-related chronic diseases, including overweight or obesity and diabetes [3-5]. Developing interventions to increase MVPA in Latina adolescents could not only improve their physical, psychosocial, and cognitive health during childhood [6] but also promote lifelong health habits that can reduce growing health disparities [7,8].

### Theory-Based Interventions to Improve PA in Latina Adults

One approach is to use theory-based interventions shown to be effective in Latina adults and adapt them to an adolescent population. We previously developed and tested the efficacy of a 6-month, culturally adapted, individually tailored, Spanish web-based PA intervention called *Pasos Hacia La Salud* [9] for Latina women based on the principles of social cognitive theory (SCT) [10,11] and the transtheoretical model (TTM) [12]. The results showed that MVPA was significantly higher in the intervention group than in the control group over the 6-month intervention [13], and the increase was maintained 6 months later [14]. In addition, the intervention group was approximately 3 times more likely to meet the national guidelines than the control group [13].

### Pilot Study to Increase PA in Latina Adolescents

On the basis of this success, we adapted the web-based intervention through formative research to make it appropriate for Latina adolescents and then conducted a 12-week single-arm pilot study (called *Niñas Saludables)* to examine the feasibility, acceptability, and potential efficacy of the adapted intervention [15]. The results of this stud*y* were promising, with 90% (19/21) of Latina girls returning for follow-up measures at the end of 12 weeks, and self-reported PA increased by 58.8 (SD 11.33) minutes per week (*P*<.001) [15]. However, acceptability was only moderate, with girls in closeout interviews expressing a desire for an intervention that was primarily delivered via mobile technologies, including social media, wearables, apps, and text messaging.

### Developing a Mobile Health Intervention

On the basis of the findings from the pilot study, a new study was launched to develop a mobile health (mHealth) multiplatform and multitechnology intervention to increase MVPA in Latina adolescents. The development of mHealth interventions requires the assimilation of user-friendly platforms and theoretical constructs in addition to technical and practical considerations and user feedback. Although mobile technologies are increasingly being incorporated into behavioral health interventions, the design of such interventions is not always guided by end users’ experience with technology [16,17]. The successful integration of technology components is most likely to occur when interventions are cocreated with behavioral scientists and stakeholders, such as members of the target population and technology industry partners. Human-centered and user-centered design emphasizes understanding the needs of a target population and developing solutions through multiple iterations of design, testing, and feedback from end users [18]; these steps have been integrated into sequential frameworks, such as IDEAS (Integrate, Design, Assess, Share) [19,20], which grounds human-centered design in behavior change theory. To ensure acceptability and usability while maintaining theoretical rigor, we chose to design the new intervention through an iterative process, with the target population guided by principles of human-centered design and the design phase of the IDEAS framework. Our ultimate goal was to work with Latina teens and tech partners to co-design mHealth intervention content that would meet the needs and preferences of the target population while being based in behavior change theory.

## Methods and Results

### Study Overview

We conducted multiple iterations of formative research partnering with Latina adolescents to co-design intervention materials for a 6-month mHealth MVPA intervention. The focus was on the design of the content and protocols rather than ideating approaches or testing efficacy; thus, the process was guided by design frameworks, including human- and user-centered design [17,18] and the four phases of the design topic area of the IDEAS framework [19].

**Figure 1:**
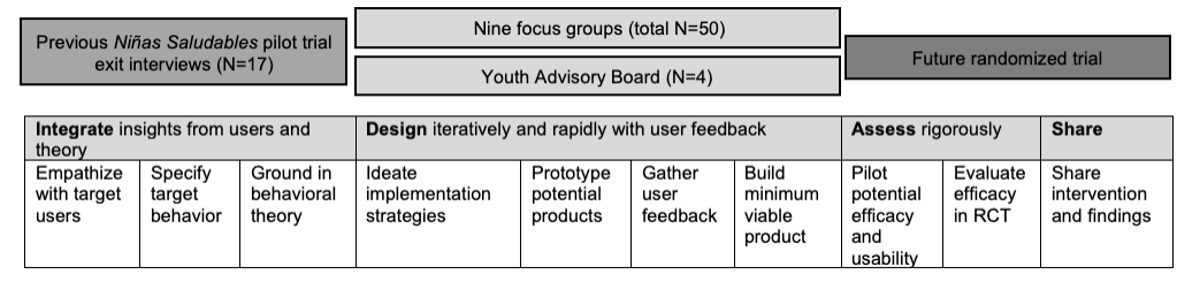
Study iterations addressing the phases of the IDEAS (Integrate, Design, Assess, Share) framework. RCT: randomized controlled trial.

The IDEAS framework [19] comprises 10 phases organized into 4 larger topic areas (integrate, design, assess, and share; Figure 1). The first area, integrate, centers on broader processes of empathizing with the target population, specifying a target behavior, and grounding in behavior change theory. This was largely addressed by the *Niñas Saludables* pilot study [15], including initial formative interviews, a single-arm pilot study, and exit interviews with participants, all of which highlighted the need for an mHealth MVPA intervention for Latina adolescents further grounded in the TTM and SCT. IDEAS is meant to act as a flexible framework and toolbox rather than a rigid structure; thus, rather than moving sequentially through all phases, we chose to conduct multiple iterations of the design phase: ideate implementation strategies, prototype potential products, gather user feedback, and build a viable minimum product. Consistent with human-centered design, we conducted multiple iterations of these four steps in collaboration with the target population. Given the novelty of this intervention approach with this population, rather than moving on to testing the new materials in a pilot trial, we chose to confirm the acceptability of the intervention materials by asking members of the target population to perform a usability test of the intervention components through an additional iteration of design feedback [17].

### Planned Intervention

The planned intervention included multiple components—an initial goal-setting session based on motivational interviewing and teaching behavior change techniques and theoretical constructs (eg, goal setting, self-monitoring, behavioral capability, and self-efficacy), followed by a 6-month intervention delivered via multiple mobile technology channels. The approach was chosen in response to feedback from the pilot study, in which girls requested more use of mobile technology; frequent, short doses of intervention (vs weekly or monthly); greater accountability and real-time feedback; and primarily visual content [15]. On the basis of this feedback, the following changes were planned for the new intervention’s content and delivery, following step 4 (ideate potential implementation strategies) of the design category:

- Theory-based behavior change tips previously posted on the website would be converted into Instagram posts.
- A commercial activity tracker (Fitbit) would be introduced to reinforce key elements of behavior change (eg, goal setting, self-monitoring, and reinforcement).
- Automated text messages would provide reminders, support, and accountability.
- The website would be redesigned to reduce text, focus on visuals, and integrate data from activity trackers.
- The study would adopt a new name, as participants felt that the name *Niñas Saluables* was more appropriate for younger girls.

The intervention content was developed through 2 phases: (1) conducting focus groups with the target population (first iteration of design) and (2) vetting the modified materials for usability with a youth advisory board (YAB) comprising Latina adolescents (second iteration). The procedure and findings are described here. As each process informed the next, the methods and results from each phase were presented contiguously.

### Focus Groups

#### Participants

Inclusion criteria for the focus groups required participants to identify as females aged between 13 and 18 years who reside in San Diego County and spoke English. We chose to develop an English intervention because the majority (88%) of Latino children in the United States speak only English or very good English, with only 1% speaking no English [21]. No interested participants were excluded because they did not speak English. Participants were recruited through in-person presentations at local middle and high schools with a high percentage of Latina students. Recruitment was also conducted through community partnerships and after-school teen programs, such as the Young Men’s Christian Association and college-readiness programs, focused on Latinas. The focus groups were described as seeking insight and feedback from teenage Latinas regarding PA and social media as a means to develop a more age-relevant and culturally relevant digital media intervention. Ultimately, the final sample of participants included 9 focus groups of 2-8 girls in each (total: N=50), aged between 13 and 18 years, with 94% (47/50) of participants identifying as Latina. Focus groups took place across San Diego County between September 19, 2019, and October 29, 2019. All study procedures and data collection materials were reviewed and approved by the University of California San Diego institutional review board, and all participants provided written informed assent or parental consent. Parental consents were available in either English or Spanish.

#### Procedures

The focus groups were structured to capture information using 2 different methods. The first portion of the focus group was formatted as a semistructured group discussion, during which the focus group moderator posed questions regarding PA likes, dislikes, barriers, and motivators as well as questions about social media apps and use.

The second portion of the focus group adopted a market research approach to assess the intervention content. Each focus group viewed 16 sample Instagram posts intended to motivate teenage girls to become more physically active, each based on a behavioral change technique [19] or SCT or TTM theoretical constructs, such as goal setting, self-monitoring, outcome expectancy, social support, or environment [10-12], and modeled off of popular Latina, fitness-focused Instagram accounts. Participants were provided with prepared notepads in which they could record their personal thoughts and reactions to each one. They were then asked to provide written feedback on 12 sample text messages based on similar constructs that included either general information (eg, “remember to schedule your activity for the week!”) or personalized information (eg, “call [friend’s name] and see if she can exercise today”). Finally, participants viewed potential study logos and potential names for the study and ranked their favorites.

Consistent with user-centered design [17], modifications were made to the content throughout the process. After 2 focus groups, staff members reviewed the feedback and eliminated posts or text messages that received consistently negative feedback. These were replaced by new samples that incorporated the suggestions.

#### Focus Group Data Synthesis

Audio-recorded data from the group discussions were transcribed and examined to identify trends in PA and social media preferences. Responses to questions regarding preferences for PA and social media were tallied to determine the most common responses. Feedback on social media posts and text messages were generally short (eg, “like it,” and “not my favorite”) and did not lend themselves to formal qualitative analysis; however, our ultimate goal was assessing end users’ experience with the intervention products, particularly whether they were perceived positively or negatively and why. Therefore, written responses to sample Instagram posts and text messages were compiled and grouped based on the nature of the feedback, with 2 staff members determining which examples were viewed positively and negatively and whether there were specific suggestions for improvement.

#### Focus Group Findings

Focus group discussions yielded trends regarding PA preferences, barriers, and motivators among the participants. When asked which activities they enjoyed the most, the top 3 responses were walking (n=11), running (n=8), and going to the gym or group fitness classes (n=7). Regarding activities they disliked, running (n=10), swimming (n=7), and biking (n=7) were the least popular. Homework (n=17) and feeling lazy or tired (n=14) were the top 2 barriers to being physically active, and family support (n=10) and supportive friends or teammates (n=9) were the most common motivators across the focus groups.

Discussions of social media use showed that a large majority of the participants favored Instagram, Snapchat, and TikTok. For Instagram, participants expressed a preference for regular posts rather than using only Instagram stories (which expire after 24 hours) because they wanted to have access to all posts on the account. Feedback on the Instagram posts revealed several patterns regarding their preferences, with the most popular posts incorporating (1) brightly colored images and limited, succinct text; (2) specific tips and applicable information on PA; (3) Latina cultural themes and Spanish phrases; (4) images of relatable and ethnically diverse models of all body types engaging in PA; (5) images promoting social support; and (6) videos demonstrating specific exercises that they could try at home. They did not like posts with darker colors, with less specific suggestions or phrases, or with images of girls with extremely thin body types.

For feedback on text messages, participants indicated the types of texts that would be most helpful and motivating as well as how frequently they should be sent. When assessing the preferences expressed by participants, there were 3 notable patterns across the focus groups. The first was a preference for messages to be sent at a rate of no more than 1 message per day. Second, on reviewing samples of both personalized and generic text messages, there was a strong preference for personalized texts that included their own name, the name of a friend, and a familiar location and a particular preference for feedback and reminders based on their own current PA rather than generic reminders. Finally, feedback revealed that texts should be casually worded with positive messages. Participants expressed antipathy toward language they perceived as “negative,” “annoying,” or “nagging,” with a particular aversion to any message that “sounds like something my mom would say.”

**Figure 2:**
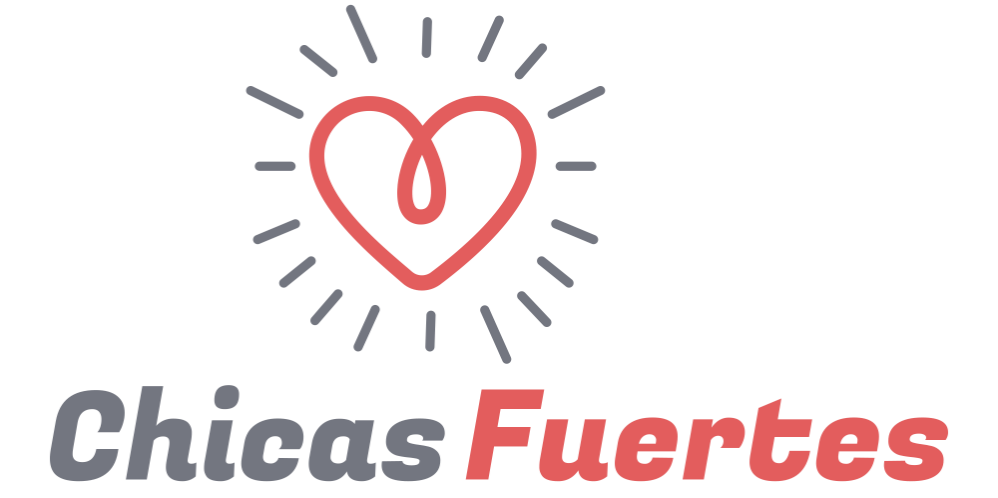
Chicas Fuertes study logo.

Finally, participants were presented 8 eight potential study names and 5 potential logo styles, and they indicated which ones they felt were most appealing and appropriate for their demographic. Then, they ranked their top 3 choices for each, with the option of creating their own idea for a study name. The most popular choice was *Chicas Fuertes*, which was selected as the new study name. Participants also expressed enthusiasm for one particular logo’s esthetic, which was chosen as the new study logo (Figure 2). Participants were also asked whether they would prefer an intervention that was only for Latinas or whether they would prefer a program targeted at adolescent girls in general that any of their friends could participate in. Participants expressed a desire for an intervention that was specifically built for and only included Latinas.

#### Incorporating Focus Group Feedback

Following the last stage of the design phase, feedback from the focus groups was used to develop a minimum viable intervention product. On the basis of the findings from the focus groups, the study adopted a new name and logo, and staff developed new materials and protocols. The new study name, *Chicas Fuertes,* and the new logo framed the study branding and were incorporated into all study and recruitment materials. Feedback on PA preferences, motivators, and barriers was factored into the development of Instagram posts and text messages to feature topics and themes that proved relevant to the study population while still being based on SCT and TTM theoretical principles.

To provide personalized text messages, we engaged tech industry partners (Fitabase, Small Steps Lab LLC) to develop and implement an algorithm to provide automated texts based on real-time data from Fitbit activity and incorporate names of family and friends the participant identified as key supporters. A text messaging schedule was developed to reflect the preferences of the participants, with messages sent 4 days per week, and the language of all text messages was worded succinctly and positively regardless of whether the message was framed to praise, reinforce, or encourage PA. For the midweek feedback text, participants who had achieved ≥45% of their weekly goal received reinforcing messages informing them that they were on track to meet their goal. Those achieving <45% of their goal received an encouraging message along with specific suggestions of behavior change techniques, social support sources, and personalized locations where they could get the rest of their activity minutes for the week. Those already meeting goals received praise and were encouraged to set a higher goal for the following week. Table 1 provides the text messaging schedule and a sample of each type of text.

**Table 1:** Sample text messages.

Day	Type of text	Example
Thursday	Midweek physical activity feedback	“So far, you’ve logged ## active minutes. Schedule time this weekend to get in some exercise with [social support #1] so you can meet this week’s goal of ## minutes by Sunday!”
Saturday	Weekly quiz reminder	“Take a 60 second break from your day and answer the weekly quiz question here! chicasfeurtessd.com”
Sunday	End of week physical activity feedback	“Here’s some good news for you! You completed your goal last week and got in ## active minutes! How many minutes will you get next week?”
Sunday	Plan activity reminder	“The best way to meet your activity goals is to plan ahead. Hop onto the website and plan your daily activity for the week! chicasfeurtessd.com”

**Figure 3:**
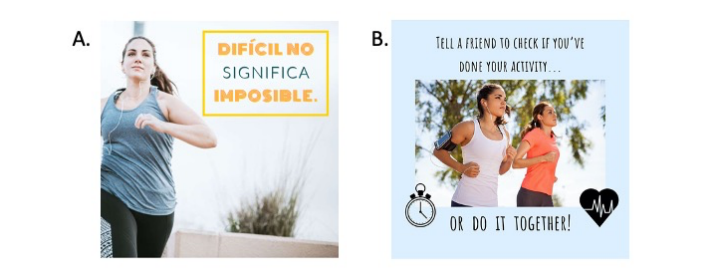
Sample Instagram posts demonstrating (A) self-efficacy and (B) social support and accountability.

When developing Instagram posts, the focus group participants’ preferences coupled with evidence-based behavior change techniques and theoretical constructs determined the nature of the content, resulting in an Instagram posting schedule of 1 post per day (Textbox 1). Each day featured a different theoretical construct or behavior change technique, including goal setting, self-monitoring, accountability, avoiding boredom, and finding time to exercise (Figure 3). Some images incorporated Spanish words or phrases, and all captions were written in English.

**Instagram posting schedule (day and behavior change construct).**

**Sunday**

- Behavior change technique

**Monday**

- Weekly challenge

**Tuesday**

- Self-efficacy

**Wednesday**

- Built environment

**Thursday**

- Modeling

**Friday**

- Benefits and outcome expectancies

**Saturday**

- Social support

Instagram story posts were also created using the polling feature for additional reminders to exercise and sync Fitbits. A study hashtag was also created to allow participants to tag their own posts if they wanted to share them with the study team or other participants.

### Youth Advisory Board

#### Participants

Following the focus groups, iterative intervention development continued with the establishment of the YAB. The purpose of the YAB was to complete another iteration of the design phase by beta testing each of the newly developed intervention components to assess usability and by providing additional ongoing feedback on the finer details of the study design to develop the final version of the intervention materials to be used in a larger trial. Recruitment for the YAB took place across local high schools and after-school programs, with teachers and leaders helping to promote the position by distributing application forms to Latina teenagers. To be eligible, participants had to self-identify as Latina, be aged between 13 and 17 years, not engage in regular PA (<150 min per week of MVPA), and express an interest in public health research and promoting health in their community. Over the course of 2 weeks, the study team received applications from 10 candidates and ultimately selected 5 based on their application responses. The 5 members came from 3 different San Diego high schools and were aged between 15 and 17 years. After 1 week, 1 member notified the study team that she was unable to fulfill her commitment, resulting in a final YAB of 4 members.

#### Procedures

The YAB members agreed to a minimum 6-month commitment, in which they would beta test intervention materials and provide honest and timely feedback on a regular basis. During months 1 to 2, the YAB was invited to follow the *Chicas Fuertes* Instagram account and provide feedback on posts developed based on the focus group feedback. Through email, the YAB provided commentary on a weekly basis on each post from the past week regarding what they liked about each post, what they did not like, and what changes they would suggest for improvement. During this time, the YAB also received sample text messages intended to provide individualized PA information and motivation and responded to each of these text messages with a detailed report of their reactions.

Beyond the scope of the focus groups, the YAB members were each given a test account for the *Chicas Fuertes* website and asked to test out all the website features during months 3 to 4. They used emails to provide comments on their experience with the website and report any bugs or confusing elements. The study website was adapted from the version used in the *Niñas Saludables* pilot study through collaboration with industry partners (Illumina Inc, Fitabase) and included resources for motivating behavior change, planning activity, and connecting with other participants. The site included a calendar for planning out weekly activity, which was overlaid with real-time activity data from Fitbits. Other features included a weekly leader board showing the 2 participants with the most active minutes that week, a message board between participants, maps of the city with links to places to be active, and tips for overcoming common barriers. Finally, the website included questionnaires used in previous trials [9] about theoretical constructs (eg, social support, self-efficacy, and processes of change) that participants would fill out monthly, also indicating their stage of change regarding PA (ie, contemplation, preparation, and action) per the TTM. On the basis of their questionnaire responses, participants received individually tailored reports on their current activity, which included their progress to date, praise for behavioral and cognitive strategies of change they used, and suggestions for areas to improve (eg, identifying sources of social support and ways to enhance self-efficacy). To keep participants engaged in the features of the website, they received points for engaging in certain activities (eg, logging in, planning their activity, and writing on the message board), and they could redeem points for prizes.

Finally, in months 5 to 6, the YAB provided feedback on finer details of the intervention and research protocols, such as the types of study participation incentives (eg, cash and gift cards) they believed would be most appealing to Latina teenagers for research participant compensation. As intervention materials and procedures continued to evolve, the YAB provided ongoing feedback. As the YAB was small (only 4 members), data were not compiled across them; rather, we focused on their individual experiences as end users of the minimum viable products.

#### YAB Findings

Intervention materials were generally perceived favorably by the YAB members, who also provided suggestions for further improvements (Table 2). Consistent with the feedback from the focus groups, YAB members enjoyed Instagram posts that were colorful and positive and particularly liked having specific suggestions or demonstrations of activity:

I liked the inspirational aspect to the post i know a lot of times when I’m on instagram and have nothing else to do and I see post with quotes that really inspire me I get up and do something active so it really helps.

YAB member 1

I looked through the Instagram feed and like it so far. The ideas that are given to encourage are actually helpful in my opinion, for instance, the post that suggests to have friends join or to dance, these I consider helpful because it’s not just hardcore Exercise like other accounts describe it to be. The videos that show certain exercises that would be easy to do at home or at a closed environment, for those who aren’t comfortable doing so outside, are also very helpful. The stories are useful, reminding to check their sync because most of people have a busy schedule so they can often forget.

YAB member 2

The post where the woman is running I didn’t like. The words were motivating yes but the color of the picture was very dark and it didn’t bring that enthusiasm for me to go for a run or something. I would have made it more clear and more.

YAB member 3

**Table 2:** Findings from the focus groups and youth advisory board.

Steps in the design phase	Findings	
	Focus groups	Youth advisory board
Prototype potential products	Built sample Instagram posts incorporating theoretical constructs Wrote sample text messages prompting goal setting and providing progress feedback Redeveloped website to be primarily visual and incorporate real-time data from wearable trackers Developed possible study logos and study names	N/A^a^
Gather user feedback	Desire for positive media; bright colors, positive wording, and encouragement versus nagging Preference for personalized (vs generic) text messages, at most 1 per day Preference for intervention exclusively for Latinas, with the incorporation of cultural themes and Spanish phrases Preference for Chicas Fuertes study name	Largely positive response to all social media posts; appreciation for mix of cognitive and behavioral strategies for behavior change; and suggestions for even more bright colors, varied body types, and Spanish phrases Positive response to text messages, suggestions to make them shorter, and appreciation for positive language Positive response to website design and content Positive response to study name and logo Preference for cash incentives and identified prizes for engagement
Build minimum viable product	Built texting algorithm for automatic goal setting and real-time feedback based on Fitbit Wrote text messages in a casual language and positive tone Developed new Instagram posts with bright colors and with models with a broad array of body types and incorporated cultural themes and Spanish phrases Developed Instagram posting schedule of 1 post per day, which were mapped onto theoretical constructs and BCTs^b^ Branded all study materials with the new study name and logo	Final schedule and content of text messages and social media posts developed and mapped onto behavior change theory constructs Bank of 120 Instagram posts developed featuring bright colors, motivational quotes, specific strategies for change, and Spanish phrases Website content finalized Text message algorithm finalized with positive, succinct text messages Prize and payment schedule finalized

^a^N/A: not applicable.

^b^BCT: behavior change technique.

As for text messages, the YAB also provided feedback on the wording, length, and content of each. For the text message “You’ve completed 38 minutes out of your goal of 100 active minutes for the week. Use the weekend to reach your goal by getting active with a friend! You can do it!!!,” YAB members provided the following feedback:

I like this message a lot actually. It’s a useful reminder and not too long and doesn’t sound like nagging.

YAB member 1 feedback

I think it might be a little wordy in the beginning but overall I think it’s pretty good. Maybe try “You’ve completed 38 out of 100 minutes for your goal this week.”

YAB member 2 feedback

For the text message “Hey girl! Got a minute? Click on chicasfuertessd.com to answer the weekly quiz question,” a YAB member provided the following feedback:

Sounds good to me! I especially like the “Got a minute” part lol.

YAB member 1 feedback

Finally, for the website, the YAB reported on the website’s esthetics, functionality, and content. Feedback was largely positive:

I really liked how everything was really accessible. Everything was really clear and aesthetically cute. I could tell on the home page that there are more things coming up since there are empty spaces. I really liked the leader board, I am the type of person that likes comparing progress and knows my level of how active I’ve been, so the leader board would really be of motivation for me. Likewise, the instagram link seems very encouraging since there is a certain display of what is in the social media page. My favorite part is that in all it seems like it’s a very supporting group to be more active, with many different ways to move along your day while being active.

YAB member 1

Some critical feedback included the 15-minute monthly questionnaire, which was too lengthy. However, the study team ultimately decided to leave this feature, as the individual tailoring of the website was based on the questionnaire responses.

The YAB also provided feedback on which prizes to offer to study participants as incentives to complete website activities. Consistently favored prizes include logo-branded items, such as a cell phone power bank, plastic tumbler, aluminum water bottle, and a Bluetooth speaker. Finally, when asked which incentives they believed would be most appealing to participants in exchange for participating in study activities (eg, attending baseline visits and completing 6-month assessments), YAB members unanimously expressed that cash or checks would be more appealing than gift cards. Findings from the focus groups and YAB are summarized in Table 2.

## Discussion

### Partnering With Stakeholders to Design Intervention Content

This paper focused on ways to address the challenges encountered by both academia and industry when creating effective, user-friendly, and culturally adapted mHealth interventions with high retention rates. The first step in developing and implementing a successful mHealth intervention is to engage a multidisciplinary leadership team comprising researchers in public health, industry partners, and the target population. For this study, we included behavioral researchers, mHealth technology partners (web developers and programmers), and multiple groups of Latina adolescents and used the principles of human-centered design and the design phase of the IDEAS framework to integrate expertise from each party.

Our previous research highlighted Latina teens’ desire for an MVPA intervention that was primarily delivered via mobile technologies. The research team ideated implementation strategies to incorporate text messaging, social media, a wearable tracker, and a redesigned website and then developed prototypes by collaborating with industry partners, including programmers and web developers. The design iterations in this study confirmed the acceptability of this approach, and preferences expressed by the target population guided the deep and surface features of the prototypes, which were mapped onto behavior change constructs from SCT and TTM. Further assessment of these prototypes through beta testing with a YAB allowed for further refinement and confirmation that the end product was viable and acceptable to the target population.

### Mobile Technology and Adolescents

Digital technologies are an attractive component of behavioral interventions because of their low cost and broad reach in both adult and pediatric populations. Access to smartphones is now nearly universal in teens (95%) and eclipses computer access (88%), and nearly all teens (91%) reported using social media [22]. However, incorporating mobile technologies into interventions has achieved mixed success [23,24]. As more digital technologies emerge, frameworks guiding the incorporation of these technologies into behavioral interventions are increasingly needed [25]. Technology will improve efficacy and reach only if it is used as a tool to meet the needs of target populations and strategically designed to facilitate, rather than compete with, elements of behavior change theory [25].

### Cocreating With Participants

Human-centered design emphasizes enlisting members of a target population as cocreators of interventions, along with refining interventions through multiple iterations of design, testing, and feedback [18]. Importantly, the IDEAS framework expands this process to center it in sound behavioral theory, along with outlining a clear sequence of steps from ideating to disseminating solutions [20]. It was developed in the process of building an app-based intervention to increase vegetable consumption in overweight adults [19], and it has also been used to develop a mobile app to reduce problem drinking among college students [26] and to build a nutrition education mobile app for families [27]. No studies have previously used it to design content for a PA intervention or an intervention specifically targeting a racial and ethnic minority population.

As previously mentioned, the framework is meant to be flexible to allow for adaptation to populations and interventions. We felt it was most important to focus on multiple iterations of the design phase of the framework rather than moving on to a pilot trial for several reasons, as follows: (1) we targeted the intervention to a niche population with unique needs and preferences based on age and ethnicity, (2) no previous MVPA interventions had specifically targeted Latina teens using digital technologies, and (3) our intervention design focused on integrating multiple technologies rather than developing a single technology channel. By cocreating the intervention content with a large sample of the target population through multiple iterations of focus groups and a YAB, we developed intervention content with high acceptability, which is ready for testing in a trial.

### Strengths

This study had several strengths. The proposed intervention focused on a key preventive health behavior (MVPA) in an at-risk, underserved population (Latina teens) and thus will fill a critical gap in public health literature. The intervention development was guided by a framework that included elements of human-centered design and behavior change theory. To ensure the production of a viable product that met the needs of the target population, we engaged in multiple iterations of feedback and design, each with a new sample from the target population. In addition, the focus groups had a large sample size (N=50).

### Limitations

Despite its notable strengths, this study had several limitations. Owing to the guidelines at schools where focus group participants were recruited from, we did not exclude non-Latina girls from participating in the focus groups. However, very few (n=3) non-Latina girls participated; thus, it is not likely that this influenced the results in any meaningful way. In addition, although the short, written feedback from participants in a market research format met the overall needs of the study, it did not allow for a more thorough qualitative analysis, which may have provided further insight into the needs and preferences of the target population. Finally, the intervention incorporated elements of SCT and the TTM because the original intervention was designed around these theories; however, other theories of behavior could have also been appropriate.

### Conclusions

We sought to design an mHealth MVPA intervention for Latina adolescents that not only incorporated digital technologies but did so in a way that was theory based and addressed the needs and preferences of our target population. Latinas were enthusiastic about a multitechnology intervention, particularly if it felt relatable, positive, and encouraging. Through multiple iterations of development and feedback, we gained insight into the needs of Latina teens and joined with industry partners to build a viable final product that is now being tested in a randomized controlled trial.

## Abbreviations

IDEAS: Integrate, Design, Assess, Share
mHealth: mobile health
MVPA: moderate-to-vigorous physical activity
PA: physical activity
SCT: social cognitive theory
TTM: transtheoretical model
YAB: youth advisory board
